# Pathways to recovery from COVID-19: characterizing input–output linkages of a targeted sector

**DOI:** 10.1186/s40008-021-00256-2

**Published:** 2021-12-23

**Authors:** Tugrul Temel, Paul Phumpiu

**Affiliations:** 1 ECOREC Economic Research and Consulting, Amsterdam, The Netherlands; 2 The World Bank, Washington, D.C , USA

**Keywords:** Input–output multipliers, Network analysis, Pathways and communities of sectors, COVID-19, Employment policy interventions, Global employment, C67, P52, J08, J21, J45, J48

## Abstract

At present, the world is facing an unprecedented employment challenge due to the COVID-19 pandemic. International Labor Organization of the United Nations expects the largest amount of youth unemployment at the global level to take place in manufacturing, real estate, wholesale, and accommodation sectors. This paper has two objectives. The first is to introduce a graph-theoretic method for identifying upstream and downstream pathways of a targeted sector and characterize them in ways that help respond to and recovery from the adverse effects of the COVID-19 pandemic. The second is to apply this method in the context of China, Japan, India, Russia, Germany, Turkey, UK and USA, which together account for about 60 percent of the world GDP. Based on the analysis of most recent input–output data from 2015, manufacturing sector is found to be top priority sector to be targeted in all the eight countries, followed by real estate and wholesale sectors, and these sectors should be coupled with isolated communities of sectors to capture external employment and growth effects. Characterizing the critical pre-COVID-19 linkages of a targeted sector should inform policy makers regarding the design of employment and growth strategies to recover from the pandemic.

## Introduction

At present, the world is facing an unprecedented employment challenge due to the COVID-19 pandemic. ILO (2020) expects the largest amount of youth unemployment at the global level to take place in manufacturing, real estate, wholesale, and accommodation sectors (see Table 1). This calls for the generation of information about the underlying properties of sectoral linkages in production networks to respond to and recover from the pandemic. This paper addresses the information need by developing a novel graph-theoretic method, which has been specifically designed to identify and characterize upstream and downstream pathways of a targeted sector in ways that help recovery from a shock to the production network. The method is applied to characterize production networks of China, Japan, India, Russia, Germany, Turkey, UK and USA, which together account for about 60 percent of the world GDP, and generate information for informed policy making to address the adverse effects of the pandemic.

**Table 1:** ILO model-based global estimates of youth employment in hard-hit sectors

Economic sectors	Baseline employment estimates for 2020 (before COVID-19 crisis)			
	Impact of crisis on economic output	Level of employment (millions)	Share in global youth employment (%)	Share of young women in total youth employment (%)
Wholesale/retail trade/repair of motor vehicles	High	74.8	17.5	41.7
Manufacturing	High	59.2	13.8	36.9
Real estate/business/administrative activities	High	16.4	3.8	43.8
Accommodation/food services	High	28.1	6.6	50.8
Transport/storage/communication	Medium–high	21.0	4.9	16.4
Arts/entertainment/recreation/other services	Medium–high	28.4	6.6	60.3
Mining/quarrying	Medium	2.9	0.7	22.6
Financial/insurance services	Medium	4.6	1.1	54.7
Construction	Medium	33.1	7.7	5.4
Agriculture, forestry and fishing	Low–medium	123.7	28.9	36.0
Utilities	Low	2.0	0.5	21.3
Public administration/defense/compulsory social security	Low	8.6	2.0	33.3
Human health/social work activities	Low	11.8	2.7	74.2
Education	Low	13.2	3.1	69.5

Source: ILO modellled estimates, November 2019

Impact ratings are based on the ILO’s assessment of real-time and financial data (ILO Monitor, released on 7 April 2020), ILOSTAT baseline data on sectoral distribution of employment (ISIC Rev. 4) and ILO Harmonized Microdata

The objective is not to estimate the output effects of the pandemic-related unemployment derived from the multiplier analysis but to elaborate on the ILO’s unemployment estimates and provide critical information that can be used in policy interventions aimed to recover from the negative output effects of the pandemic. For example, ILO forecasts a substantial amount of unemployment in the manufacturing sector due to the COVID-19 related loss of working hours, while our paper generates information from the past input–output data that can be employed in the design of policy interventions aimed to minimize or avoid such unemployment, and thereby offer ways to recover the projected output lossssuch information is the identification of significant upstream and downstream pathways of the targeted manufacturing sector; a second piece of information is the specific grouping of sectors supporting the manufacturing sector (i.e., community structure of the manufacturing sector); a third piece of information is the critical binary links connecting several communities (i.e., betweenness) when the manufacturing sector is targeted. Knowledge of these network relations centered around the manufacturing sector can be used to formulate employment and growth strategies.

The empirical analysis uses IO data from 2015, which is the most recent available data in OECD database. Therefore, our paper assumes that the properties of a production system in 2015 of a country remained unchanged during the period 2015–2020. The employment strategies elaborated in what follows should be interpreted relative to the 2015 IO properties of the country examined. The findings show that manufacturing (MA2) is top priority sector to be targeted in all the eight countries, followed by real estate (EST) and wholesale (WHS) sectors, and that these sectors should be coupled with isolated communities of sectors to capture external employment effects from the interacting communities (or clusters). Naturally, sector coupling would vary across countries, depending on the linkages between the communities identified.

This paper is organized in five sections. Following the Introduction, Section 2 presents a brief review of the literature to position and motivate the current paper, pointing out, where it contributes to the literature by developing a new method for characterizing a targeted sector with its upstream and downstream pathways.

Section 3 describes the new method and the three network concepts used in the analysis. Section 4 applies the method using the 2015 IO data for eights countries. Drawing on the results from Section 4, Section 5 discusses how to integrate the new information obtained from partial sectoral analysis into wider employment policy interventions. Section 6 concludes the paper.

## From a single sector to a network of sectors

To date, single sector analysis has received more attention compared to networked-sector analysis, undermining the importance of the inter-sector connectivity in a production network. A key sector, for example, is usually identified based on the size of its output multiplier or of its backward and forward multipliers. The premise is that the larger its multiplier is, the larger its impact would be. However, the assessment of the impact of a sector would make more sense if its position and role within the network it belongs to is considered. That is, the size of its multiplier as well as its connectivity to the rest of the network provide complementary information useful for the impact assessment. The method we develop in this paper has three specific objectives. The first is to identify key upstream and downstream pathways centered around a targeted sector. The second is to derive communities (or clusters) of sectors of the given targeted sector and their within-community interaction patterns. The third is to identify the critical between-community linkages transmitting external influence from one community to another. These objectives shift the focus from individual sectors to pathways of key upstream and downstream sectors and their community structure. In other words, we do not concentrate on a single key sector or few sectors but rather characterize the dominant production relations arising when an individual sector is targeted. The objectives stated above highlight this point by emphasizing the application of graph-theoretic concepts, such as connectivity, community structure, connected components, and source–sink pathways. An important point is that the analysis is carried out for a targeted sector, which allows for a cross-country comparison of dominant patterns of linkages when the same sector is targeted across different countries.

Our method enriches the multiplier analysis often carried out in the literature by using graph-theoretic concepts and methods. In that sense, key sector identification based on output multipliers, for example, is implemented by using graph-theoretic methods based on vertex centrality measures. The importance of a sector is assessed not only by the size of its multipliers but also by its positional superiority within a narrowly defined network. In doing so, the method exploits the structure of connectivity of a sector or a community of sectors. In the context of input-output analysis, the existing literature calls a sector to be key if it has the largest output multiplier in the Leontief inverse matrix or if it concurrently has the largest backward and forward multipliers. Our method, however, would define a sector as key in the context of the sectoral connectivity implied by the targeting algorithm developed. This would imply that sector *i* that may be key in the case of targeting sector *j* may not be key when sector *k* is targeted. Take, for example, an IO matrix with five sectors {**A, B, C, D, E**} with **A** being the key sector in terms of output multiplier and **D** being the non-key sector. If the objective is to create the largest impact on sector **C** from **A** and the leading pathway is $\mathbf {A}\rightarrow \mathbf {B}\rightarrow \mathbf {D}\rightarrow \mathbf {C}$, the most critical sector becomes sector **D** since the absence of **D**, no matter how high its multiplier is, reduces the entire pathway to nothing. That is, the weakest linkage in a functionally connected pathway represents the highest degree of success in achieving the final objective, which is to increase the impact on sector **C**. With this example, the focus shifts from identifying the key sector(s) to identifying the key pathway(s). In doing so, the meaning of the term “key” also changes from a single sector to communities of the sectors along the key pathways in which all the sectors are functionally (or algorithmically) linked. A “star” network illustrated in Fig. 5 with the weakest sector **MA2** being at the center and other sectors becoming the satellites of **MA2** is a good example of sector **MA2** becoming the most critical sector in the network, although it may very well be a non-key sector in terms of output multiplier. The removal of **MA2** from the network leads to the collapse of the star network.

In the literature of complex networks, the concept of cascading behaviour is used to refer to influence subgraphs in which state of certain vertices influences the behaviour of others.^1^ Formally, an “infection” event can spread contagion through infected players which constitute a propagation tree, known as a cascade. In fact, our method is very similar to the cascading concept used to identify certain patterns of linkages in a production network in which a given sector is targeted. The cascading in our algorithm starts with a targeted sector. In the first step, the immediate input providers of the targeted sector are identified. In the second step, the input providers of the immediate input providers of the targeted sector are identified and so on. This results in a subgraph incorporating the upstream linkages of the targeted sector. Likewise, the algorithm also derives the downstream cascading of the output supply of a targeted sector. Once identified, the upstream and downstream cascades are combined. The cascade structure accommodates nonlinearity of the relations, while stressing the functional connectivity of the sectors (Kleinberg 2013; Taglioni and Winkler 2016).

In the development of the method, some ideas from key sector identification (Schultz 1977), structural path analysis (Defourny and Thorbecke 1984), fundamental economic structures (Hewings et al. 1989; Jensen et al. 1991), and interconnectedness in regional input–output matrices (Lantner and Carluer 2004) have been exploited to characterize upstream and downstream production pathways of a targeted sector. The method combines backward and forward linkages to create a network in which both demand and supply information flows between sectors. The Leontief inverse of the IO matrix measures the level of backward linkages measured as the proportion of total output that represents purchases from sectors in an economy. Hirschman (1977) defines forward linkage of a particular sector as the proportion of total output of this sector that does not go to final demand but to other sectors. Following Dietzenbacher (1997), the Ghosh matrix represents forward multipliers as a measure of change in output values in response to changes in the prices of primary inputs.

Loviscek (1982) suggests the use of both backward (input demand) and forward (output supply) linkages in order to obtain an accurate picture of interindustry structure as such linkages incorporate demand-side and supply side information. In case of targeting **A**, for example, our method identifies the pathways and their communities incorporating input providers to sector **A** (i.e., upstream to sector **A**) and consumers of outputs of sector **A** (i.e., downstream to sector **A**). In the sense of Loviscek (1982), combining demand and supply-side information, our method characterizes a unified production network of sector **A** in which **A**’s input demand and output supply can be examined simultaneously by considering its demand and supply constraints.

Jensen et al. (1991)’s concept of fundamental economic structure (FES) relates to our work. In a spatial context, IO cells containing flows that are consistently present at predictable levels over a range of economies are called “fundamental” as they represent economic activities inevitably required in all economies. Other IO cells with data for more region-specific sectors (for example, mining) define the nonfundamental economic structure (NFES). The identifiable patterns/linkages of predictable cells constitute a FES, which can be estimated using regression techniques. Our method, however, offers a graph-theoretic approach to revealing key FESs by targeting a given sector over a time-series of IO matrices. For example, one may target sector **A** by using a time-series of IO matrices and discover the FESs as the pathways or community structures that remain unchanged over a relative long period of time. For purposes of illustration, the current paper applied the method to a time-series of IO matrices (11 IO matrices during 2005–2015) of China by targeting the same sector **MA2** at the same threshold level (0.15 < *x*). The findings confirm that there is a fundamental network that remains unchanged over the period 2005–2015 in China.^2^

## Method

### Upstream and downstream linkages of a targeted sector

**Fig. 1:**
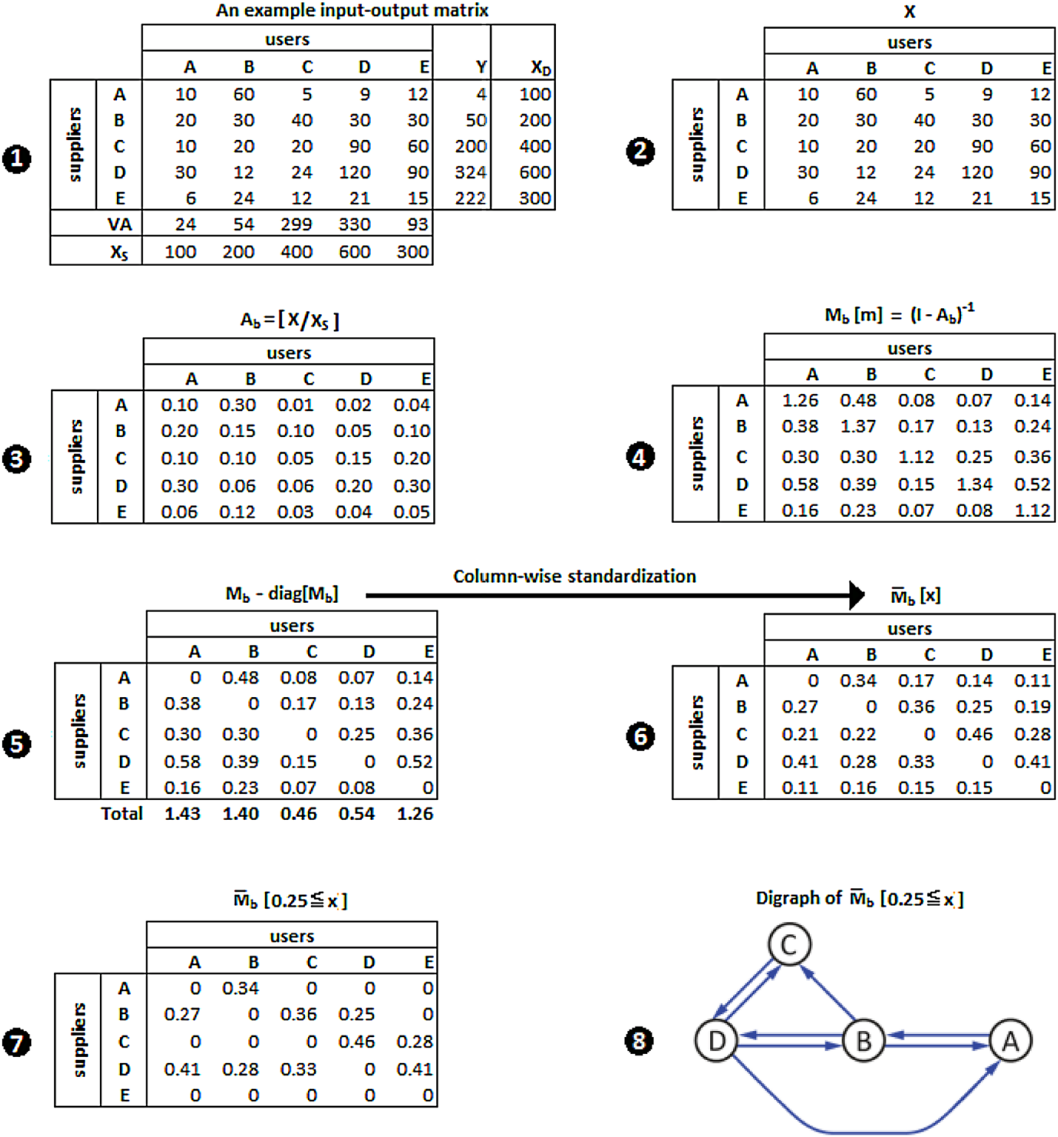
Identifying upstream linkages of a targeted sector **A**

An Algorithm is developed that aims to identify upstream and downstream linkages of a targeted sector, and its implementation is illustrated within the input-output (IO) framework. For purposes of simplicity, an example IO matrix given in Fig. 1(1) is used that allows for the demonstration of the step-by-step implementation of the algorithm. The example IO matrix consists of five components. The first component is an intermediate consumption sub-matrix (**X**) in Fig. 1(2) with five sectors: {**A**, **B**, **C**, **D**, **E**}. The second is a column-vector of final consumption (**Y**); the third, a column-vector of total demand ($\mathbf {X}_{D}$); the fourth, a row-vector of value-added (**VA**); and the fifth, a row-vector of total supply ($\mathbf {X}_{S}$), all of which are illustrated in Fig. 1(1). Sub-matrix **X** in Fig. 1(2) and total output supply $\mathbf {X}_{S}$ is used to calculate the backward technical coefficients matrix, $A_{b}=[\mathbf {X_{ij}}/\mathbf {X_{S}^{j}}]$, given in Fig. 1(3). The Leontief inverse matrix, $\mathbf {M}_{b}[m]\equiv (I-A_{b})^{-1}$, in Fig.  1(4) defines the so-called backward multiplier matrix with *m* denoting individual multipliers, where *I* stands for an identity matrix with dimension (5, 5). For notational simplicity, we will use $\mathbf {M}_{b}$. In order to focus on the analysis of inter-sectoral connectivity, the diagonal cells in $\mathbf {M}_{b}[m]$ are replaced with zeros; that is, $\mathbf {M}_{b}-diag[\mathbf {M}{}_{b}]$ in Fig.  1(5).^3^ The matrix, ${\overline{\mathbf {M}}}_{b}$, in Fig.  1(6) is obtained through column-wise standardization of $\mathbf {M}_{b}-diag[\mathbf {M}{}_{b}]$. In doing so, individual multipliers of a user sector are adjusted to reflect the relative importance of a supplier in the output multiplier of the user sector. The standardized matrix ${\overline{\mathbf {M}}}_{b}[x]$ is the only input used in targeting a sector by setting an arbitrary threshold significance level (${\overline{\mathbf {M}}}_{b}(0.25\leqslant x)$) with *x* being matrix elements greater than or equal to 0.25. The matrix ${\overline{\mathbf {M}}}_{b}(0.25\leqslant x)$ in Fig. 1(7) is a reduced form of ${\overline{\mathbf {M}}}_{b}[x]$, which includes only the cells greater than or equal to 0.25. Suppose that a user sector **A** is targeted to identify the entire chain of its direct and indirect suppliers; that is, to identify the entire chain of upstream sectors of user **A**.

**Fig. 2:**
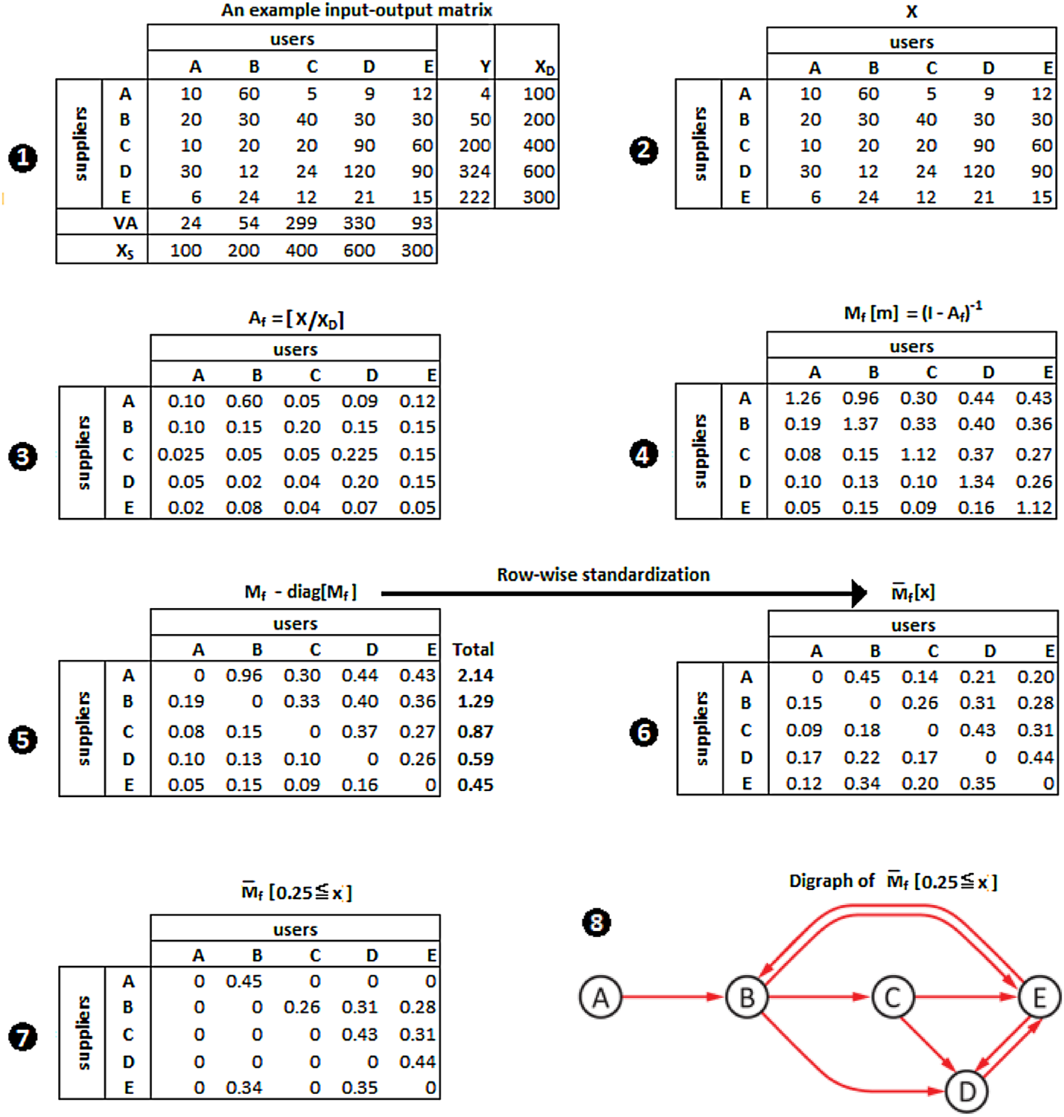
Identifying downstream linkages of a targeted sector **A**

Using backward multipliers in $\mathbf {M}_{b}$ represents only half through the targeting exercise, because a backward linkage defines the input linkage of the targeted sector. To be complete, other half should be based on forward multipliers in $\mathbf {M}_{f}[m]\equiv (I-A_{f})^{-1}$ (the so-called Ghosh matrix) given in Fig. 2(4) as a forward linkage defines the output linkage of the targeted sector. For notational simplicity, we will use $\mathbf {M}_{f}$. The only difference between the derivation of backward multipliers and forward multipliers is that the latter uses the forward coefficients matrix, $A_{f}=[\mathbf {X_{ji}}/\mathbf {X_{D}^{j}}]$, in Fig. 2(3) to calculate the row-wise standardized matrix, ${\overline{\mathbf {M}}}_{f}[x]$, in Fig. 2(6). The matrix ${\overline{\mathbf {M}}}_{f}(0.25\leqslant x)$ in Fig.  2(7) is a reduced form of ${\overline{\mathbf {M}}}_{f}$, which includes only the cells greater than or equal to 0.25. Suppose that a supplier sector **A** is targeted to identify the entire chain of its direct and indirect users; that is, to identify the entire chain of downstream sectors of supplier **A**.^4^

Having derived the backward and forward reduced forms, ${\overline{\mathbf {M}}}_{b}(0.25\leqslant x)$ in Fig.  1(7) and ${\overline{\mathbf {M}}}_{f}(0.25\leqslant x)$ in Fig.  2(7), the next step is to use them to identify the upstream and downstream pathways of a targeted sector, for example, **A**, and map these pathways as a single network with a view to examining the connectivity of the upstream and downstream sectors of the targeted sector **A**. Replicating the targeting exercise for the rest of the sectors in the IO matrix would generate five networks, one for each sector. In what follows, we explain the implementation of the algorithm developed in three steps.^5^

**Fig. 3:**
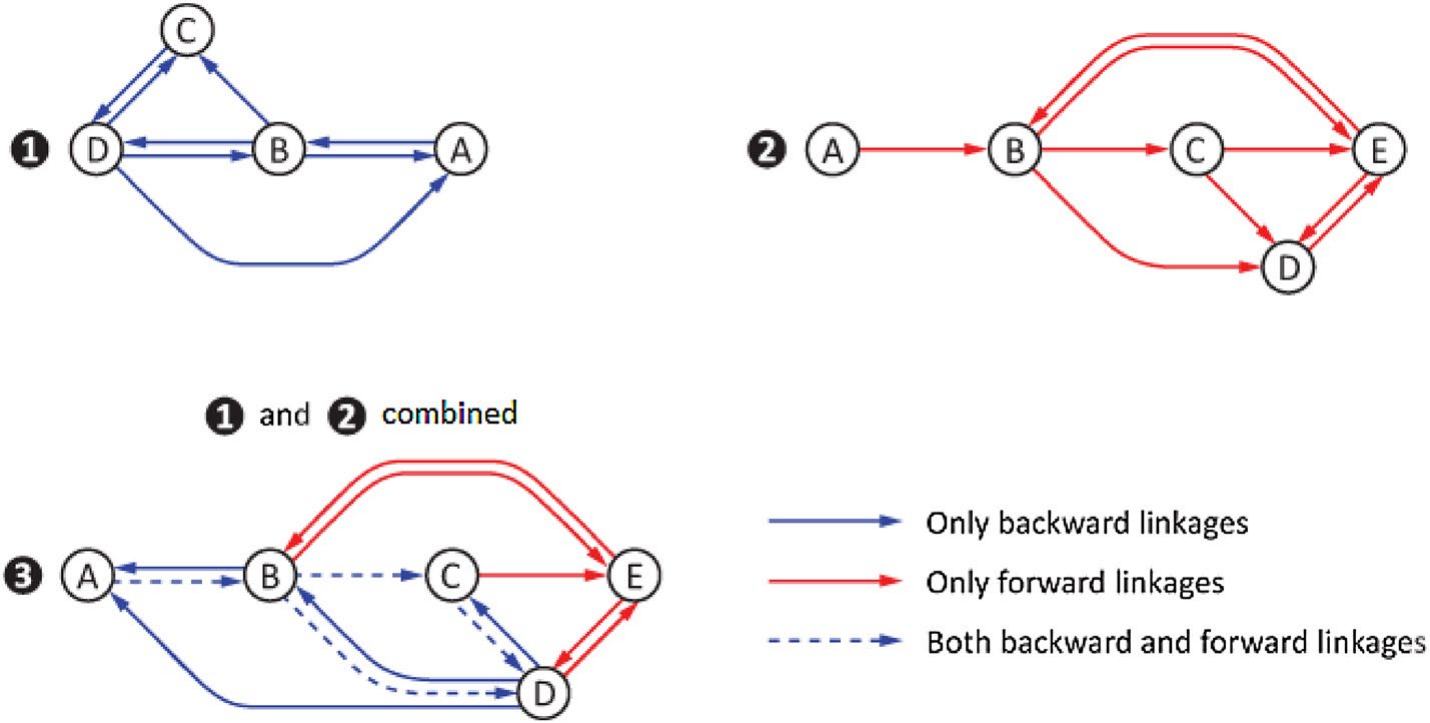
Combined network of upstream and downstream linkages of a targeted sector **A**

- Step 1: (using ${\overline{\mathbf {M}}}_{b}(0.25\leqslant x)$: At an arbitrarily set significance level, 0.25, from input side, we target user sector **A** associated with the 1st column of ${\overline{\mathbf {M}}}_{b}(0.25\leqslant x)$. This means that those numbers which are equal to or greater than 0.25 in the 1st column are considered as significant enough from the user perspective, in which case there are two significant linkages. One is from **B** to **A** with a coefficient of 0.27 (denoted as $B\rightarrow A$), and another is from **D** to **A** with a coefficient of 0.41 (denoted by $D\rightarrow A$). Then, moving to the 2nd column associated with user sector **B**, we observe that **A** also provides input to **B** (denoted by $A\rightarrow B$) with a strength level of 0.34, and that **D** provides input to **B** (denoted by $D\rightarrow B$) with a strength level of 0.28. We then move on to identify the significant suppliers of user sector **D** associated with the 4th column. Suppliers **B** and **C** provide input to user **D** through the two linkages denoted by $B\rightarrow D$ and $C\rightarrow D$ with the strength levels of 0.25 and 0.46, respectively. Finally, we identify suppliers of user sector **C** by moving to the 3rd column, in which case suppliers **B** and **D** are observed as significant with the strength levels of 0.36 for the linkage $B\rightarrow C$ and 0.33 for the linkage $D\rightarrow C$. This completes the search of significant direct and indirect suppliers of the targeted user sector **A**. Important to note is that, although the IO matrix has five sectors, the search for the suppliers of user **A **results in a directed network of four sectors, revealing that sector **E **is irrelevant from the point of input supply to the targeted sector **A**. Combining all of the binary linkages identified in this step generates the directed network in Fig. 1(8), which consists of a set of eight binary linkages when user sector **A** is targeted:
  1 $$\begin{aligned} Input=\{B\rightarrow A,\, D\rightarrow A,\, A\rightarrow B,\, D\rightarrow B,\, B\rightarrow D,\, C\rightarrow D,\, B\rightarrow C,\, D\rightarrow C\}. \end{aligned}$$
- Step 2: (using ${\overline{\mathbf {M}}}_{f}(0.25\leqslant x)$: At the same significance level, 0.25, from output side, we target supplier sector **A** associated with the 1st row of ${\overline{\mathbf {M}}}_{f}(0.25\leqslant x)$. This means that those numbers which are equal to or greater than 0.25 in the 1st row are considered as significant enough from the supplier perspective, in which case there is one significant linkage from **A** to **B** with the strength level of 0.45 (denoted as $A\rightarrow B$). Then, moving to the 2nd row associated with supplier sector **B**, we observe three linkages from **B**: $B\rightarrow C$ with a strength level of 0.26, $B\rightarrow D$ with a strength level of 0.31, and $B\rightarrow E$ with a strength level of 0.28. We then move on to identify the significant users of supplier sector **C** associated with the 3rd row. Supplier **C** provides output to users **D** and **E**, which are, respectively, denoted by $C\rightarrow D$ and $C\rightarrow E$ with the strength levels of 0.43 and 0.31. Supplier **D** associated with the 4th row provides output to user **E **(denoted by $D\rightarrow E$) with the strength level of 0.44. Finally, supplier **E** associated with the 5th row provides output to users **B** and **D**, which are denoted by $E\rightarrow B$ and $E\rightarrow D$ with the strength levels of 0.34 and 0.35, respectively. This completes the search of significant direct and indirect users of the targeted supplier sector **A**. Combining all of the binary output linkages identified in this step generates the directed network in Fig. 2(8), which consists of a set of nine binary linkages when supplier sector **A** is targeted:
  2 $$\begin{aligned} Output=\{A\rightarrow B,\, B\rightarrow C,\, B\rightarrow D,\, B\rightarrow E,\, C\rightarrow D,\, C\rightarrow E,\, D\rightarrow E,\, E\rightarrow B,\, E\rightarrow D\}. \end{aligned}$$
- Step 3: It should be noted that, as illustrated in Fig. 3(3),*input* network in 1 and *output* network in 2 have four common linkages given in 3:
  3 $$\begin{aligned} Input\,\cap \, output=\{A\dashrightarrow B,\, B\dashrightarrow C,\, B\dashrightarrow D,\, C\dashrightarrow D\}, \end{aligned}$$
  which simultaneously carry both input (denoted by solid blue arrows) and output (denoted by solid red arrows arrows). These common linkages are shown by dashed blue arrows in Fig.  3(3).

To sum up, when sector **A **is targeted, its upstream linkages form the input supply network shown in Fig.  3(1), whereas its downstream linkages form the output supply network shown in Fig.  3(2). As seen in Fig.  3(3), the two networks combined fully characterize sector **A**’s connectivity (i.e., all the linkages that matter for **A** at the given threshold strength level of 0.25) both in input and output space.

### Connected components and their communities

Any digraph such as the one illustrated in Fig.  3(3) can be further analyzed by deriving its connected components and community structures. A directed graph is said to be connected if there is a path between all pairs of vertices (or production sectors in our context). A connected component of a digraph is a maximal connected sub-graph. Connected components of a directed graph comprise an acyclic directed graph, meaning that individual connected components form a partition into sub-graphs that are themselves connected.

**Fig. 4:**
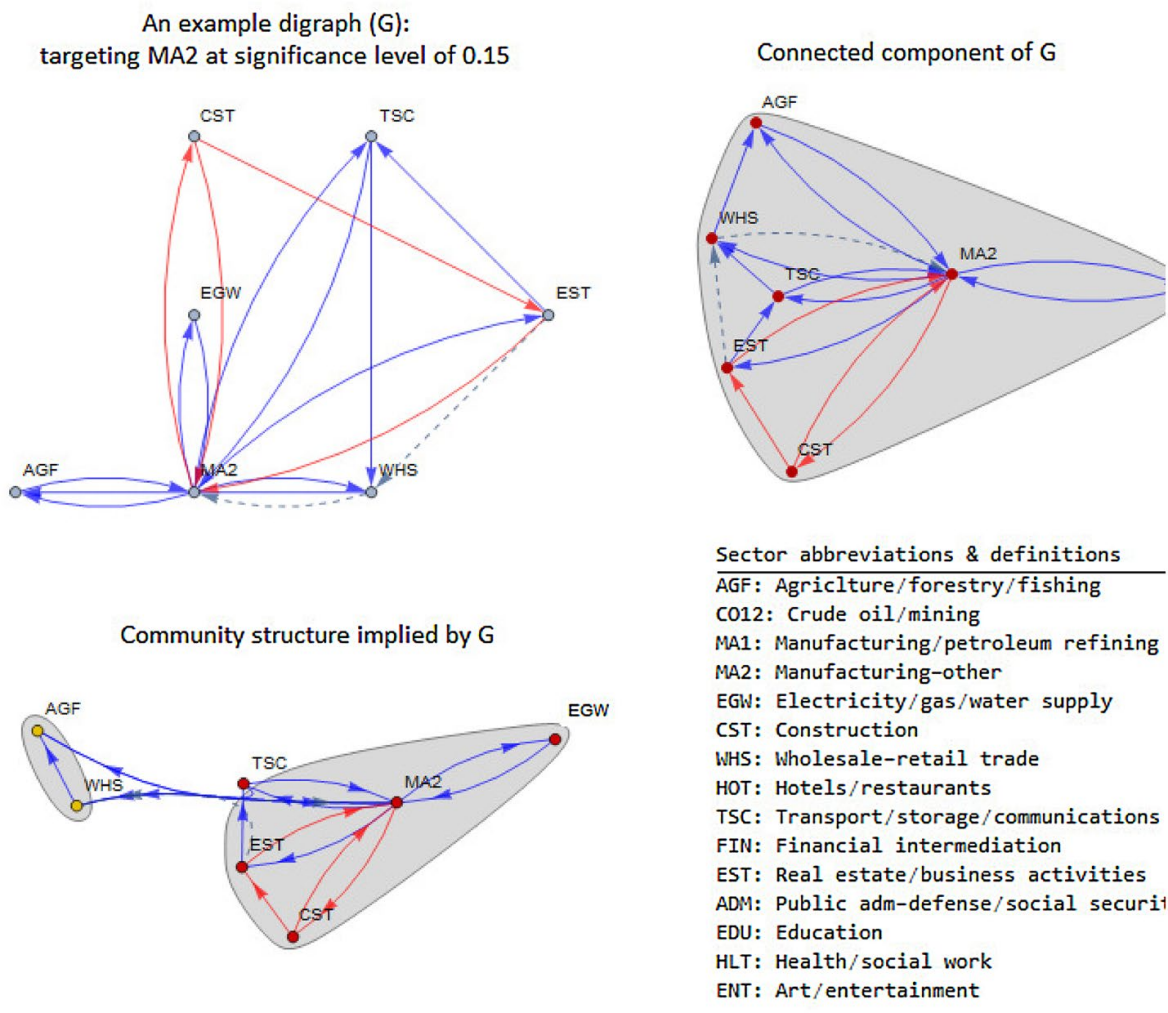
Example digraph **G**, its connected components and community structure

To visually illustrate these concepts, a digraph **G** with 15 sectors (nodes) is used as an example (see Fig. 4). The digraph **G** has a single connected component with 7 sectors out of 15. Since the connected component is a single entity within which all sectors are linked to each other, any influence exerted on a sector will flow across all the sectors within the component. There is no way for a sector to avoid the impact on itself of others within the component as they are all connected.

In the next step, the question is whether there is a partition of a connected component into sub-graphs, each one of which maximizes Modularity statistic (Charikar 2000; Fortunato et al. 2004; Newman and Girvan 2004; Capocci et al. 2005; Newman 2006, 2009; Easley and Kleinberg 2010; Fortunato 2010; Giatsidis et al. 2011). We know that sectors within a connected component are all linked, but we do not know whether there are distinct sub-graphs within the connected component concerned. The community structure of the connected component is detected on the basis of Community Modularity statistic. The detected community structure tells us that there are two communities (or clusters) of sectors, {AGF, WHS} and {TSC, EST, CST, MA2, EGW}, that are highly correlated or homogenous in terms of Modularity criterion, centrality measure for example (see Fig. 4).

### A network of key sectors

**Fig. 5:**
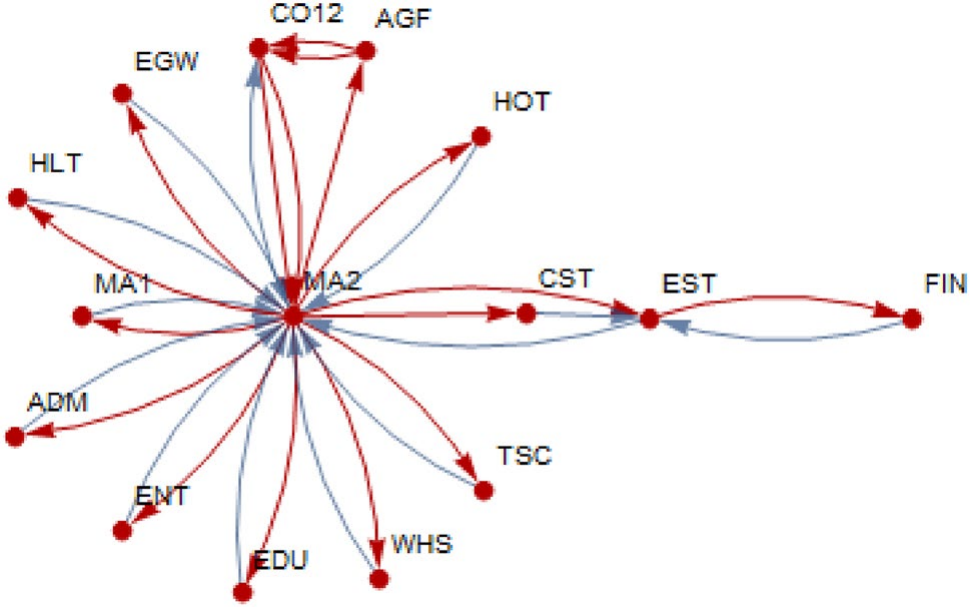
Network of key sectors from both backward and forward linkages

From a sectoral perspective, a sector is said to be key to another sector if it has the maximum contribution to the total output multiplier of the other sector. From an economy-wide perspective, however, a sector is said to be key if its total output multiplier is the largest compared to the total output multipliers of other sectors in the economy. We adopt the sectoral perspective and separately identify the key sectors from a backward multiplier matrix and those from a forward multiplier matrix. Then, we construct a directed graph using the pooled set of linkages obtained from the backward (blue arrows) and forward (red arrows) multiplier matrices. The final directed graph illustrated in Fig. 5 represents a combined network consisting of the most influential linkages (blue and red arrows combined) on the input and output sides.

For simplicity, we examine the case in which a sector has one key input (output) sector ($k=1$) only, meaning that the maximum backward (forward) multiplier is selected from each column (row) in a backward (forward) multiplier matrix. This yields two directed graphs: one for backward linkages (blue) and another for forward linkages (red). Thereafter, the two graphs are combined to generate the final network of input–output linkages of key sectors with $k=1$. The same procedure can be applied for $k>1$, depending on the size of the multiplier matrix examined. The choice of *k* is arbitrary, depending on the objective pursued.

As illustrated in Fig. 5, from the network perspective, **MA2** stands alone as a critical sector as it has the function of coordinating changes in the rest of the network. Almost all sectors in the network are linked to **MA2**, making this sector so powerful for the survival of the network. Removing it from the network will lead to the collapse of the entire network. In this sense, **MA2** is a key sector. This interpretation emphasizes not only the importance of connectivity but also the network structure.

## Implementation

### Data: input–output matrices

The method and the network concepts described in Section 3 are applied to characterize IO systems of eight countries: China, India, Japan, Russia in Asia; Germany, Turkey and UK in Europe, and USA. The IO data used in the implementation are obtained from OECD’s IO database for the most recent available year 2015.^6^ The OECD IO matrices with 36 sectors have been aggregated to 15 sectors using the 2008 UN definitions for sector aggregation (United Nations, Development and Bank 2009). The aggregation allows for a comparative analysis of the IO systems across countries. Our point of departure is the sector aggregation of ILO. The first column in Table 2 shows the individual sectors in OECD IO database; the second column shows the aggregated sectors used in this study; and the third column shows the ILO aggregation of 14 sectors. A slight difference between our aggregation and ILO’s aggregation comes from the fact that we disaggregated “Manufacturing sector” (which is modeled by ILO as a single sector) into two sub-sectors: **MA1** in our analysis covers the petroleum and refinery activities, while **MA2** captures the rest of the manufacturing activities conducted in the manufacturing sub-sectors. **MA2** is an important sector for all the countries examined in this study as it represents the agglomeration of several inter-connected industrial sectors. Bilateral linkages between manufacturing and the service sectors, including wholesale, retail, finance, real estate, hotels-tourism, etc. are important, and in this study, we aim to pay more attention to the output and employment effects created through the linkages concerned.

**Table 2:** Sector aggregation

Sectors in the OECD Input–output matrices	Sector aggregation in this study	ILO sector aggregation
TTL_01T03: Agriculture/forestry/fishing	AGF: Agriculture, forestry and fishing	Same as this study
TTL_05T06: Mining/extraction of energy products	CO12: Crude oil/mining	Mining and quarrying
TTL_07T08: Mining/quarrying of non-energy products	MA1: Manufacturing/petroleum refining	
TTL_09: Mining support service activities		
TTL_10T12: Food products/beverages/tobacco	MA2: Manufacturing-other	Same as this study
TTL_13T15: Textiles/wearing apparel/leather & related products		
TTL_16: Wood/products of wood and cork (except furniture)		
TTL_17T18: Paper products and printing		
TTL_19: Coke and refined petroleum products		
TTL_20T21: Chemicals and pharmaceutical products		
TTL_22: Rubber and plastics products		
TTL_23: Other non-metallic mineral products		
TTL_24: Manufacture of basic metals		
TTL_25: Fabricated metal products, except machinery/equipment		
TTL_26: Computer, electronic and optical products		
TTL_27: Electrical equipment		
TTL_28: Machinery and equipment n.e.c.		
TTL_29: Motor vehicles, trailers and semi-trailers		
TTL_30: Other transport equipment		
TTL_31T33: Other manufacturing/repair and installation of machinery and equipment		
TTL_35T39: Electricity/gas/water supply/sewerage/waste/ remediation services	EGW: Electricity/gas/water supply	Same as this study
TTL_41T43: Construction	CST: Construction	Same as this study
TTL_45T47: Wholesale/retail trade; repair of motor vehicles	WHS: Wholesale–retail trade	Same as this study
TTL_55T56: Accommodation and food services	HOT: Hotels/restaurants	Same as this study
TTL_58T60: Publishing/audiovisual/broadcasting activities	TSC: Transport/storage/communication	Same as this study
TTL_49T53: Transportation and storage		
TTL_61: Telecommunications		
TTL_62T63: IT and other information services		
TTL_64T66: Financial and insurance activities	FIN: Financial intermediation	Same as this study
TTL_69T82: Other business sector services	EST: Real estate/business activities	Same as this study
TTL_84: Public administration/defense/compulsory social security	ADM: Public adm./defense/social sec.	Same as this study
TTL_85: Education	EDU: Education	Same as this study
TTL_86T88: Human health and social work	HLT: Health/social work	Same as this study
TTL_90T96: Arts/entertainment/recreation & other services	ART: Art/entertainment	Same as this study
TTL_97T98: Private households with employed persons		

Concerning youth unemployment due to COVID-19, ILO’s global estimates conjecture that manufacturing (**MA2**), wholesale and retail (**WHS**), real estate (**EST**), and accommodation (**HOT**) sectors will be hit hard (see Table 1 on page 8 of ILO (2020)), which is the point of departure for the analysis conducted in this paper. It should be noted that the sample of the eight countries accounts for a substantial portion of the world GDP, and hence there is the need for developing strategies to avoid the bleak unemployment picture projected by ILO. The analysis of the current paper should provide critical information for use in the effective design of policy interventions targeting the four sectors. Government policies targeting employment in the hard-hit sectors should be informed of the characteristics of the backward and forward linkage structures of these sectors.

### Sector targeting

The method developed is applied to target the four sectors identified by ILO (2020). If, for example, sector *i* is targeted for policy intervention, we first need to identify input suppliers of that sector, then identify input suppliers of sector i’s input suppliers, followed sequentially by the identification of other input suppliers. This chain of backward linkages between the targeted sector and its first degree, second degree, third degree etc. input suppliers would show the network of upstream linkages of the targeted sector with the rest of the production system. The chain of linkages from the rest of the system to the targeted sector will fully identify the target sector’s production dependencies. Likewise, the targeted sector is also characterized with respect to the type of consumers (both intermediate and final) of its commodities. We first need to identify the critical buyers (sectors) of the commodities produced by the targeted sector, and then sequentially identify the buyers of the commodities produced by the buyers of commodities of the targeted sector and so on. This type of downstream linkages would show how the target will be affected by changes in the demand for its commodities. With this type of forward sectoral links, we would characterize the commodity demand network of the targeted sector. Together, a combined map of backward and forward input–output flows from the perspective of the targeted sector will help us uncover the critical sectoral pathways of linkages which are most important for the performance of the targeted sector.

The empirical analysis is based on a given threshold significance level of a multiplier. This level is set to be 15 percent, meaning that the analysis carried out considers those multipliers having an explanatory power of 15 percent or higher out of the total input/output multiplier of the sector targeted. The linkages shown represent those linkages accounting for 15 percent or more of the multipliers influencing the targeted sector.^7^

In case of ${\underline{\mathbf{targeting \,\,\mathbf{MA2} }}}$, an interesting pattern of input-output flows arises across the countries examined. In four countries in Asia, agriculture (**AGF**), crude oil and mining (**CO12**), and WHS sectors supply significant *input*; in two European countries, financial business (**FIN**), transportation-storage-communication (**TSC**) and **WHS** sectors transfer significant *input*; in Turkey, electricity-gas-water (**EGW**) and **HOT** sectors reveal significant *input* flows; and in USA, interestingly, the composition of the critical input suppliers includes **AGF**, **CO12**, **FIN** and **TSC**, which is “almost” the union of the critical sectors in Asia and Europe. With respect to *output* flows, we observe that construction (**CST**) and **EST** sectors unanimously arise as critical sectors whose outputs are demanded in the rest of the economy. Concerning sectoral dependencies, we observe that {**CO12**, **CST**, **EST**, **WHS**, **MA2**} reveal strong dependencies. **EST** is vitally important to control the changes in the rest of the economies of Japan, Russia, Germany, UK, Turkey and USA. Of these six countries, USA, UK and Russia reveal a much stronger dependency structure implied by a large number of sector linkages. For example, in USA, we have the dependency structure of:

$$\begin{aligned} \mathbf {EST}\dashrightarrow \mathbf {WHS}\,\,\, and\,\,\,\mathbf {EST}\dashrightarrow \mathbf {MA2}. \end{aligned}$$

In UK, the dependency structure is of:

$$\begin{aligned} \mathbf {CST}\dashrightarrow \mathbf {EST}\dashrightarrow \mathbf {WHS}\dashrightarrow \mathbf {MA2}\dashrightarrow \mathbf {CST}, \end{aligned}$$

and in Russia, it is:

$$\begin{aligned} \mathbf {EST}\dashrightarrow \mathbf {WHS}\dashrightarrow \mathbf {MA2}\,\,\, and\,\,\,\mathbf {WHS}\dashrightarrow \mathbf {CO12}\dashrightarrow \mathbf {MA2}. \end{aligned}$$

The larger the number of linkages, the higher the complexity of dependency, and the more challenging will be to design policy interventions that involve multiple sectors.

In case of ${\underline{\mathbf{targeting \,\,\mathbf{WHS} }}}$, a similar pattern of linkages arises across the countries examined. In Asian countries, **AGF**, **CO12** and **MA2** supply significant *input*; in two European countries, **FIN**, **MA2** and **TSC** transfer significant *input*; in Turkey, sectors **EGW**, **HOT** and **MA2** reveal significant *input* flows; and in USA, the composition of the critical input suppliers includes **AGF**, **CO12**, **FIN** and **TSC**, which is “almost” the union of the critical sectors in Asia and Europe. With respect to *output* flows, we observe that **CST**, **EST** and **MA2** play a critical role in all countries. Concerning **sectoral dependencies**, we observe that China and India do not show any sector dependencies, whereas others show varying degrees of dependencies among {**CO12**, **CST**, **EST**, **MA2**}. The highest degree of dependency is observed in UK, with a pathway:

$$\begin{aligned} \mathbf {CST}\dashrightarrow \mathbf {EST}\dashrightarrow \mathbf {WHS}\dashrightarrow \mathbf {MA2}. \end{aligned}$$

This suggests that before targeting **WHS**, the implications on **WHS** of a change in **CST** and **EST** should be analyzed as the performance of **WHS** is strongly dependent on the type of changes in **CST** and **EST**. Russia is also facing somewhat weaker dependency, with a pathway:

$$\begin{aligned} \mathbf {EST}\dashrightarrow \mathbf {WHS}\dashrightarrow \mathbf {CO12}\dashrightarrow \mathbf {MA2}. \end{aligned}$$

In case of ${\underline{\mathbf{targeting \,\,\mathbf{EST} }}}$, similarities exist among Asian countries and USA. **AGF**, **CO12**, **MA2** and **WHS** play an important role in *input* supply; in Germany and UK, **FIN** and **TSC** still represent the core of input supply. Turkey reveals structural differences compared to other countries, in which case **EGW**, **HOT** and **MA2** supply critical amount of input to the rest of the economy. What is interesting in the case of Turkey is that the publicly managed **EGW** and private sector **HOT** occupy a central place in input supply, but these sectors play no role in input supply in the other six countries examined. With this feature, Turkey is distinguished from the other six countries. Concerning *output* supply, except UK and Germany, **CST** and **MA2** unanimously arise as two critical sectors whose outputs are consumed by others. Regarding **sectoral dependencies**, China, India, Germany and USA show no dependency, while others show dependency involving **WHS**.

In case of ${\underline{\mathbf{targeting \,\,\mathbf{HOT} }}}$, the results look very similar to the case in which **EST** is targeted. Four Asian countries have the same sectors {**AGF**, **CO12**, **MA2**, **WHS**} significant in input supply; two European countries share commonality but Germany has a wider input supply network {**FIN**, **MA2**, **TSC**, **WHS**} compared to UK having two input supply sectors {**FIN**, **TSC**}. USA shows a combination of Asian and European networks, including {**AGF**, **CO12**, **FIN**, **MA2**, **TSC**, **WHS**}. Turkey is distinguished with a very different set of input suppliers, including {**EGW**, **MA2**}. Regarding *output* supply, except UK and Germany, **CST**, **EST**, and **MA2** represent the core of output suppliers in Japan, India, Russia and Turkey, while **CST** and **MA2** represent the core suppliers in China and USA. With respect to **sectoral dependencies**, **EST** and **WHS** constitute the core of dependencies, which is extended by **CST**, **CO12**, and **MA2** in Russia and UK.

### Connected components and community structures

**Fig. 6:**
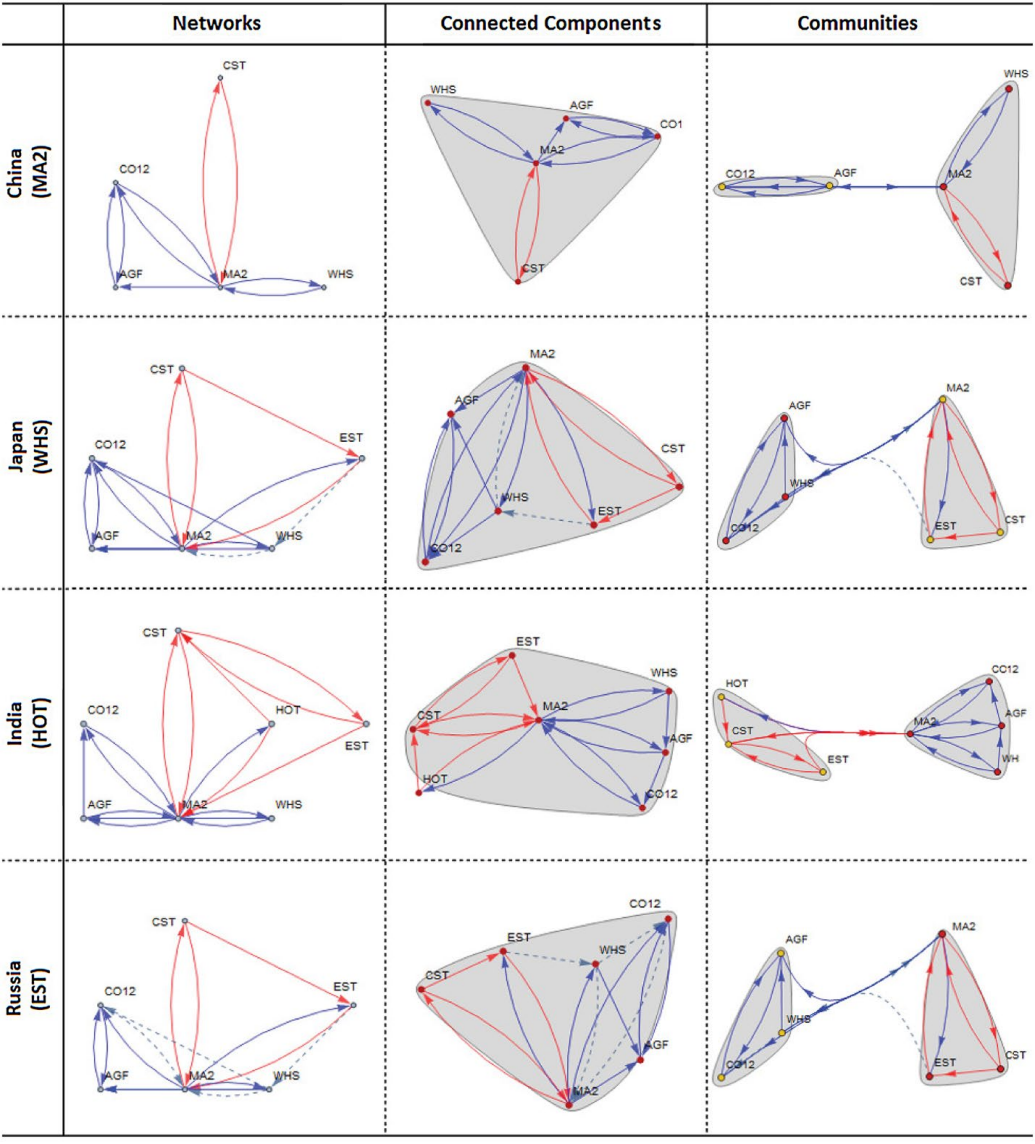
Selected sectors targeted at significance level of 0.15 in China, Japan, India and Russia

**Fig. 7:**
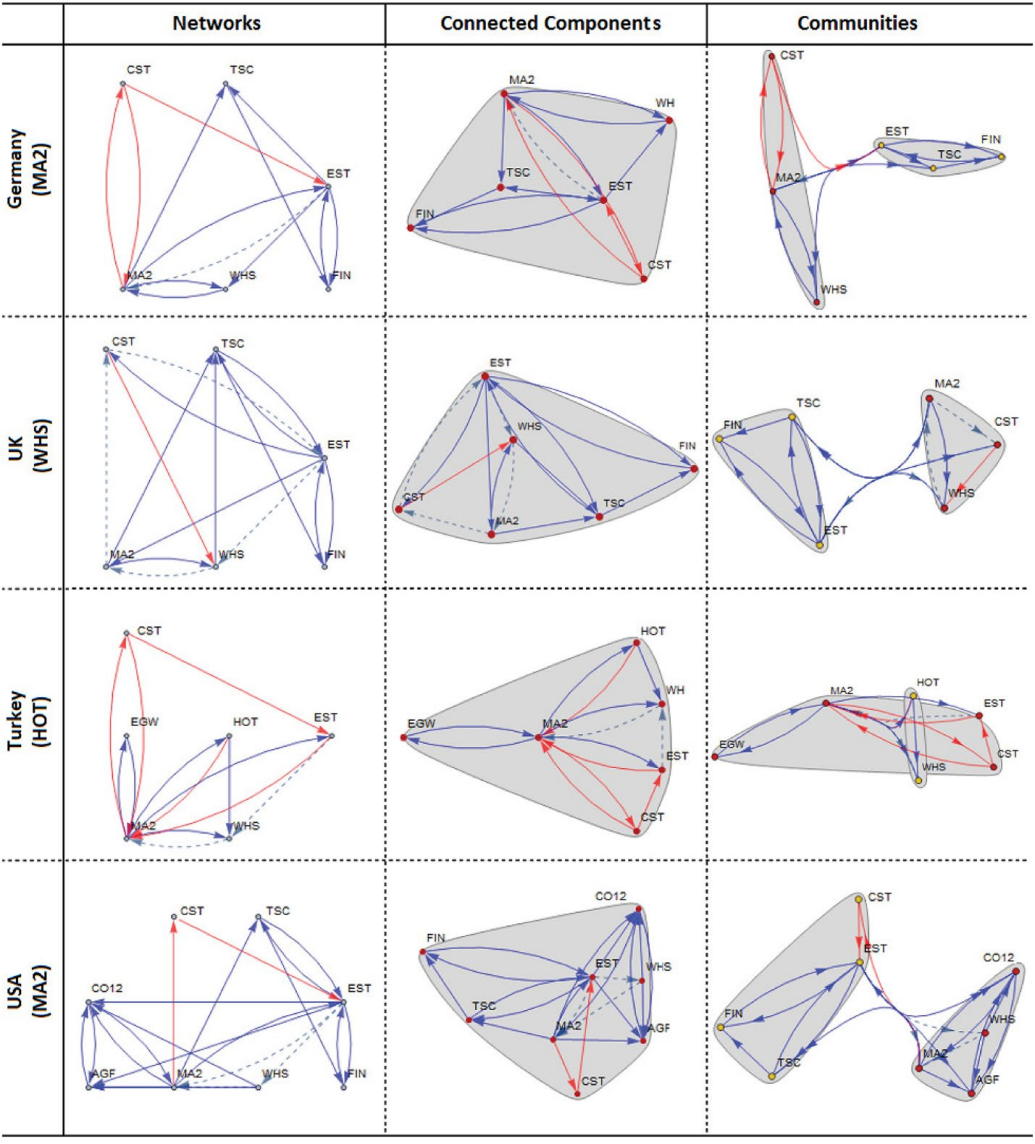
Selected sectors targeted at significance level of 0.15 in Germany, UK, Turkey and USA

Drawing on the targeting-based networks across countries (see the 1st column of Figs. 6 and 7),^8^ all of the IO systems examined show only one connected component (see the 2nd column of Figs.  6 and 7). This finding suggests that the networks shown in the 1st column are all connected, implying that a change in input supply or output supply of a sector will be transmitted to the rest of the network through either direct or indirect linkages. Any intervention to a single sector within the connected component will have repercussions in the rest of the network. However, the level of repercussions would vary across sectors in the network depending on the size of multipliers associated with each linkage.

A deeper analysis of a connected component is to search for communities (or clusters) within the connected component examined. Community analysis aims to detect partitions of the connected component in such a way as to reflect potentially different repercussions within each partition (or community). The community structures identified for each connected component are presented in the 3rd column of Figs. 6 and 7. The mapping of the communities identified shows that almost all connected components across countries and sectors have two communities (or clusters). In a more detailed policy design, each community should be individually targeted as a group as its members show similarity with respect to network betweenness centrality criterion.^9^ It is also critical that policies should aim to strengthen the linkages connecting the two communities to maximize the overall benefits from the connectivity of the communities. Otherwise, positive externalities that may arise from one community will not be captured by policy interventions.

Three constructs stand out for use in the design of policy interventions: (i) directed graphs describing input and output flow structure implied by targeting a specific sector, (ii) the underlying dependency pathways, and (iii) the key sectors that ensure the highest benefit from interactions in a network. Take, for example, Germany. It is characterized by the network of upstream and downstream pathways, simple dependency, $\mathbf {EST}\dashrightarrow \mathbf {MA2}$, and key sectors {**EST**, **MA2**}. The first construct produces all the relevant pathways of sectors from/to the targeted **MA2**. The second suggests that, no matter which sector is targeted, **MA2**’s performance strongly depends on the input and output of **EST**. The third construct is that these sectors are key as they have not only the largest multiplier values but also occupy the critical position in the network. In the case of UK, a very complex pathway arises:

$$\begin{aligned} \mathbf {CST}\dashrightarrow \mathbf {EST}\dashrightarrow \mathbf {WHS}\dashrightarrow \mathbf {MA2}\dashrightarrow \mathbf {CST}, \end{aligned}$$

in which case **CST** plays a key role both as a source of policy change and as a sink of the impact of the change concerned (i.e., a loop starting from a change in **CST** and ending with an effect on itself). The fact that it is a closed loop makes it challenging to control the changes along the chain of linkages, **EST**$\dashrightarrow$**WHS**$\dashrightarrow$**MA2**, because this two-edge pathway represents a constraint for **CST**. When, for example, **WHS** is targeted, its impact on **CST** as well as **CST**’s impact on **WHS** via changes in **EST** must be considered because **WHS** is a member of the closed loop. The other countries can be analyzed in a similar fashion at will.

**Fig. 8:**
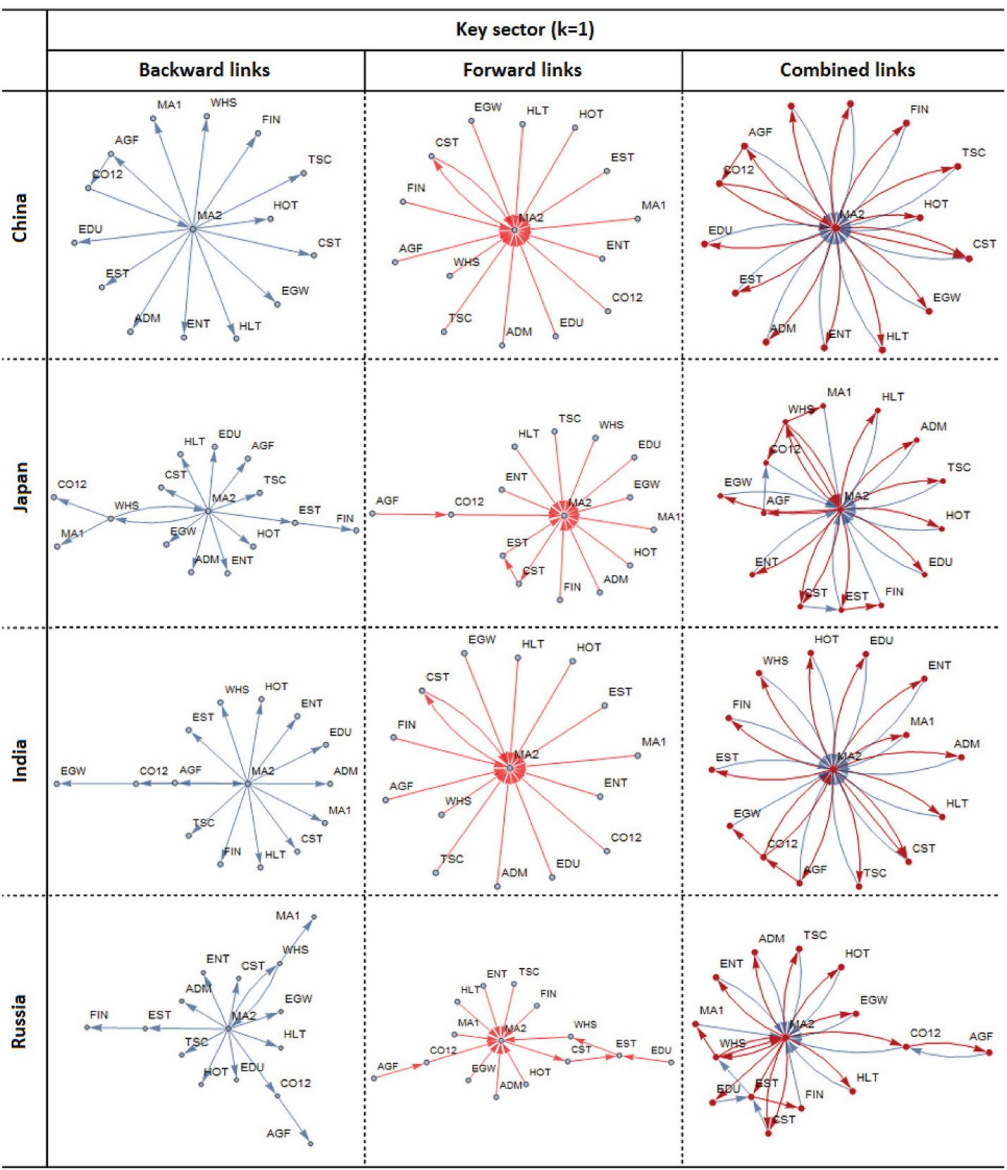
Key sectors of the economy (1)

**Fig. 9:**
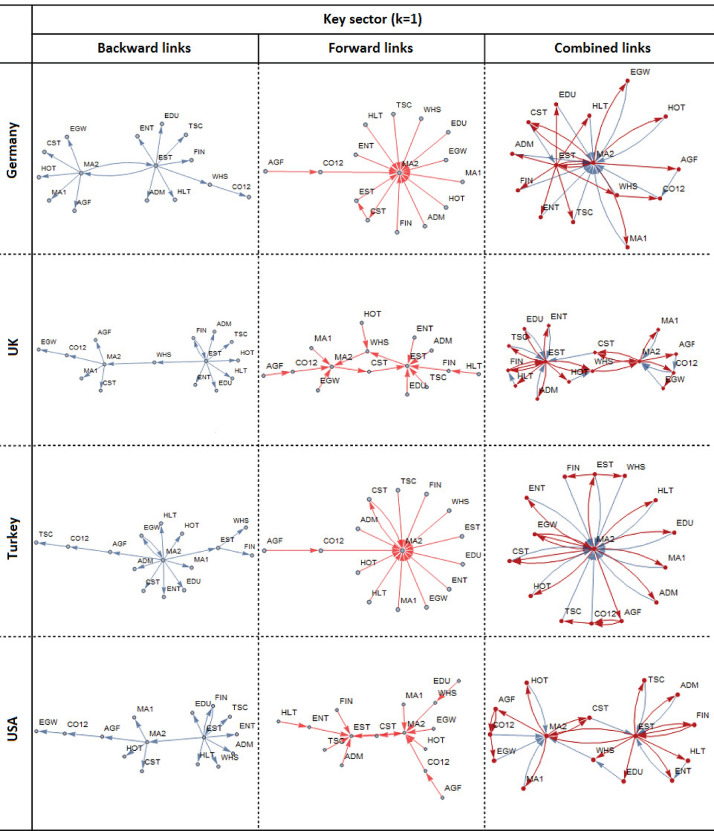
Key sectors of the economy (2)

For each country, we also identified key sectors in the sense described in Section 3.3 (see Figs. 8 and 9). **EST** and **MA2** are identified as key sectors in Germany, USA, Turkey, and UK; **MA2** and **WHS** are key sectors in Japan and Russia; and **MA2** is key for China and India. Apparently, there is some kind of homogeneity in the maximum multiplier sectors across the countries. Across all the countries analyzed, **MA2** is the key sector to be targeted to generate the maximum employment and output through its multiplier effects as well as its connectivity to the rest of the economy.

## Discussion of the findings

Drawing on the findings elaborated in Section 4, we suggest ways to achieve the best employment and output outcomes at the country level. The key to success lies in ensuring that each country prioritizes the identified critical sectors, while considering community structures and pathways of sector dependencies as constraints of policy interventions. In other words, we propose to formulate an employment and growth strategy as a constraint optimization problem, the objective of which is to maximize output of a targeted sector(s) subject to sector specific as well as structural constraints, including the degree of sector connectedness, community structure (size and density), and pathways of sectoral dependencies. In what follows, we elaborate on how to employ the information generated in the formulation of policy interventions.

First, the domain of any policy targeting with a view to ensuring the pre-COVID-19 employment level should necessarily include {**AGF**, **CO12**, **CST**, **EST**, **FIN**, **MA2**, **WHS**, **HOT**}, in which case {**EST**, **MA2**} are the core sectors with the largest multiplier effects and critical connectivity patterns both in input and output markets. Together, these cores would act as catalyst for the growth in other sectors through the input–output linkages.

Second, in all the eight countries examined, except for USA, the policy intervention networks are composed of two communities (or clusters). Knowledge of the characteristics (i.e., number of sectors, their interactions, and linkage density) of the community structures identified should be utilized in employment policy design. In China, {**CST**, **MA2**, **WHS**} and {**AGF**, **CO12**} represent the two robust core communities reflecting the strongest linkages among its members, and these communities survive no matter which sector is targeted. This suggests that the highest gain in employment in China can be materialized by exploiting the linkage properties within individual communities, as well as the linkage strength between the communities.

In Japan, there are two robust core communities, {**CST**, **EST**, **MA2**} and {**AGF**, **CO12**}, no matter which sector is targeted. Interestingly, members of the first community are linked to each other in output markets, while members of the second community interact only in input markets. This makes the targeting easier and more appealing. It is easier in the sense that if employment creation is targeted in output markets, the interactions among sectors in the first community should be examined; if, however, employment in input markets is targeted, then the interactions among sectors in the second community should be analyzed. It is appealing, because the sectors, where the final impact of targeting is expected are isolated in two different communities, because these communities are connected through the linkages in input markets only.

In India, there are two robust core communities, {**CST**, **EST**} and {**AGF**, **CO12**, **MA2**, **WHS**}, no matter which sector is targeted. Members of the first community are linked to each other in output markets, while members of the second community are linked only in input markets. Similar to the case of Japan, targeting is easy and appealing. It is easy in the sense that if employment creation is targeted in output markets, the interactions among sectors in the first community should be examined; if, however, employment in input markets is targeted, then the interactions among sectors in the second community should be analyzed. It is appealing, because the sectors, where the final impact of targeting is expected are isolated in two different communities. Interestingly, the linkages between the two core communities are all about the interactions in output markets only, as opposed to the Japanese case in which the communities are linked through input market linkages.

In Russia, there are two robust core communities, {**CST**, **EST**, **MA2**} and {**AGF**, **CO12**, **WHS**}. Members of the first community are linked to each other in both input and output markets, while members of the second community interact only in input markets. The two communities are linked through the input linkages only. If employment is targeted independent of market type, the first community should be examined; if, however, employment is targeted in input markets, the second community should be analyzed. These communities are linked in input markets, because they are connected through the linkages in input markets only.

The two EU countries, Germany and the UK, share commonalities, while showing key differences from the Asian countries, including China, Japan, India and Russia. Both Germany and the UK have two identical communities: {**EST**, **FIN**, **TSC**} and {**CST**, **MA2**, **WHS**} when **EST**, **MA2** and **WHS** are targeted. In both countries, the first community arises in input markets, while the second community has linkages in both input and output markets. The type of linkages connecting the two communities is different across Germany and the UK, however. In Germany, the two communities are connected through linkages both in input and output markets, while in the UK through input market linkages only. Germany and the UK show stronger differences when sector **HOT** is targeted. The communities differ both in terms of sector composition and the type of linkages connecting the communities. Therefore, **HOT** needs special attention when policies are designed to promote employment in this sector.

The U.S. shows characteristics that have commonalities both with the Asian and the EU countries. Two robust communities, {**AGF**, **CO12**, **MA2**, **WHS**} and {**CST**, **EST**, **FIN**, **TSC**}, arise when **EST**, **MA2** and **WHS** are targeted. The first community consisting of only input linkages is similar to the Asian case, while the second one consisting of both input and output linkages is similar to the EU case. These communities are connected through input and output linkages. The picture becomes quite different when **HOT** is targeted. Three communities emerge, two of which {**AGF**, **CO12**, **WHS**} and {**EST**, **FIN**, **TSC**} are all about input linkages, and the third one {**CST**, **MA2**, **HOT**} has mixed linkages. This reflects different dependency structure **HOT** has with the rest of the economy.

Finally, Turkey shows a completely different linkage structure between two core communities: {**HOT**, **WHS**} and {**CST**, **EST**, **EGW**} no matter which sector is targeted. The first community is all about input linkages, while the second is mixed with input and output market linkages. These communities are also linked with mixed linkages. What is interesting and important is to observe **EGW** to play a significant role in the core economic activities. This observation is unique to Turkey as **EGW** has not been observed as critical in the other 7 countries examined.

A third suggestion is that knowledge of the critical binary sectoral links ensuring cross-community connectedness is essential for informed policy interventions. The policies aimed to ensure the continuity of cross-community links should be integrated into wider economic policies to materialize potential employment benefits from the interactions between the communities. The potential gains from such connectedness will be forgone if the policies implemented dismantle or do not consider the connectedness of the existing communities. For example, in China, the connectedness of the two communities discussed above requires the presence of at least one linkage out of two: {(**MA2**, **AGF**), (**MA2**, **FIN**)}; in Japan, the presence of at least one linkage out of four: {(**AGF**, **EST**), (**AGF**, **HOT**), (**WHS**, **MA2**), (**WHS**, **CO12**)}, and so on. When there are more than two communities, which is the case in USA, then at least three linkages must be present to tie all the communities together.

To sum up, the implementation of the algorithm and the findings are neither final nor complete. The results are reflecting only part of the big picture as they are conditional to the threshold significance level chosen. The study elaborates on ways to provide policy guidance based on the results obtained. A general policy recommendation based on the results is that coupling the targeted sector with its key partners should be the way forward to reap the full benefits of policy interventions. Such interventions should also exploit patterns of linkages between the targeted sector and its community in the production system.

## Conclusions

An unprecedented, COVID-19-driven unemployment challenge is addressed using network analysis of input–output matrices of eight countries, including China, Japan, India, Russia, Germany, Turkey, UK and USA. A novel algorithm is developed to identify critical input–output backward and forward linkages of a targeted sector. Based on these linkages, sectoral dependencies and pathways of sectoral interactions are characterized to generate critical information that is needed for the design of employment policy interventions.

Using concepts from network analysis and OECD input–output data, this paper develops an algorithm to uncover critical patterns of sector linkages and features of country-level production systems. In order to respond to the projected COVID-19-related youth unemployment in manufacturing, real estate, wholesale and accommodation sectors, the paper produces information that can be used in employment strategy development in the context of the eight countries analyzed, which together account for about 60 percent of the world GDP. Employment strategy development is discussed with the help of a constrained optimization problem, the objective of which is to maximize employment under sector and production system constraints. The empirical configuration of sectoral pathways of interactions, sectoral input–output dependencies, and sectoral communities defines the domain of the constraints for optimal employment. Broad elements of an optimal employment strategy is then elaborated using this configuration. Manufacturing is found to be top priority sector to be targeted in all the eight countries, followed by real estate and wholesale sectors, and these sectors should be coupled with isolated communities of sectors to capture external employment effects.

Needless to say, the closed-economy analysis carried out in this paper presents an incomplete picture of the actual employment possibilities as the current analysis does not take into account the employment creation effects of the trade-linkages across countries. Adopting an open-economy framework, future research should incorporate the sectoral production linkages among trading countries, and in doing so, potential international sources of employment creation in a given country can be explored. Such information would provide input to the design of evidence-based trade and employment policy. OECD multi-country input–output matrices are available to conduct the type of open-economy employment analysis we are advocating here.

## Data Availability

The input–output data for eight countries examined in this paper are publicly available at https://stats.oecd.org/Index.aspx?DataSetCode=IOTSI4_2018. Furthermore, the data will be available upon request.

## Footnotes

1 The reader is referred to (Easley and Kleinberg 2010; Borge-Holthoefer et al. 2013) for further reading on cascading behaviour in complex socio-technical networks.

2 The FES issue is beyond the scope of this paper; however, it should be pointed out that the analysis of FESs in a country can be easily done by applying our method. In fact, we applied it to China for the period of 2005–2015, and the visual network structures that remain unchanged have been found. The results would be available upon request.

3 The diagonal elements of the multiplier matrix are intentionally set to be equal to zero, in order to focus on the inter-sectoral connectivity. An empirical regularity is that a very large majority of IO multiplier matrices are diagonally dominant as their diagonal multipliers are larger than one. This is also the case for IO multiplier matrices of the countries examined in this paper. The reason is that a sector produces part of its total input demand in addition to the production of inputs demanded by the rest of the sectors in the economy. Miller and Blair (2009, pp. 90–96) explain this within inter-regional IO framework, and Henderson and Evans (2017) explains the same issue with an example IO matrix (see https://www.fwrc.msstate.edu/pubs/implan_2017.pdf).

4 The reader is referred to Miller and Blair (2009) for an extensive description of how to use input–output matrices in policy analysis.

5 The *Algorithm* has been developed by the authors. *Mathematica* Code developed at https://mathematica.stackexchange.com/questions/210169/how-can-i-generate-a-tailor-made-directed-graph-from-a-given-matrix has been adjusted to perform the computations in this paper. The adjusted *Algorithm* will be available upon request. Special thanks go to Mathematica expert recognized in Mathematica forum as @KGLR.

6 see https://stats.oecd.org/Index.aspx?DataSetCode=IOTSI4_2018 for OECD input–output data for 64 countries over 11 years from 2005 through 2015.

7 The figures and tables presenting the results were kept at a minimum due to the space limitation.

8 It should be noted that targeting exercise was conducted for four sectors across eight countries but we only presented a single targeting for each country. The targeting networks not presented here will be made available upon request.

9 The Girvan–Newman algorithm is applied to identify communities. This algorithm first identifies edges in a network that lie between communities and then removes them, leaving behind just the communities themselves. The algorithm employs the graph-theoretic betweenness centrality measure, which assigns a number to each edge which is large if the edge lies “between” many pairs of nodes.

## Declarations

### Disclaimer

The findings and interpretations expressed in this paper are entirely those of the authors. They do not necessarily reflect the view of the World Bank, its executive directors, or the countries they represent.
